# Effects of Gelatin Hydrogel Containing Anti-Transforming Growth Factor-β Antibody in a Canine Filtration Surgery Model

**DOI:** 10.3390/ijms18050985

**Published:** 2017-05-05

**Authors:** Michiko Maeda, Shota Kojima, Tetsuya Sugiyama, Denan Jin, Shinji Takai, Hidehiro Oku, Ryohsuke Kohmoto, Mari Ueki, Tsunehiko Ikeda

**Affiliations:** 1 Department of Ophthalmology, Osaka Medical College, Takatsuki-City, Osaka 569-8686, Japan; opt182@osaka-med.ac.jp (M.M.); tsugiyama@osaka-med.ac.jp (T.S.); opt025@osaka-med.ac.jp (H.O.); ryousuke0218@hotmail.co.jp (R.K.); opt089@osaka-med.ac.jp (M.U.); tikeda@osaka-med.ac.jp (T.I.); 2 Nakano Eye Clinic of Kyoto Medical Cooperative, Kyoto 604-8404, Japan; 3 Department of Innovative Medicine, Osaka Medical College, Takatsuki-City, Osaka 569-8686, Japan; pha012@osaka-med.ac.jp (D.J.); pha010@osaka-med.ac.jp (S.T.)

**Keywords:** trabeculectomy, glaucoma, gelatin hydrogel, transforming growth factor-β, beagles

## Abstract

In this present study, we investigated the effect of a controlled release of anti-transforming growth factor β (TGF-β) antibody on intraocular pressure (IOP), bleb formation, and conjunctival scarring in a canine glaucoma filtration surgery model using gelatin hydrogel (GH). Glaucoma surgery models were made in 14 eyes of 14 beagles and divided into the following two groups: (1) subconjunctival implantation of anti-TGF-β antibody-loaded GH (GH-TGF-β group, *n* = 7), and (2) subconjunctival implantation of GH alone (GH group, *n* = 7). IOP and bleb features were then assessed in each eye at 2- and 4-weeks postoperative, followed by histological evaluation. We found that IOP was significantly reduced at 4-weeks postoperative in the two groups (*p* < 0.05) and that IOP in the GH-TGF-β-group eyes was significantly lower than that in the GH-group eyes (*p* = 0.006). In addition, the bleb score at 4-weeks postoperative was significantly higher in the GH-TGF-β group than in the GH group (*p* < 0.05), and the densities of fibroblasts, proliferative-cell nuclear antigen (PCNA)-positive cells, mast cells, and TGF-β-positive cells were significantly lower in the GH-TGF-β group than in the GH group. The findings of this study suggest that, compared with the GH-group eyes, implantation of anti-TGF-β antibody-loaded GH maintains IOP reduction and bleb formation by suppressing conjunctival scarring due to the proliferation of fibroblasts for a longer time period via a sustained release of anti-TGF-β antibody from GH.

## 1. Introduction

Glaucoma filtration surgery (i.e., trabeculectomy) is a primary treatment for glaucoma that results in decreased intraocular pressure (IOP) by draining the aqueous humor to the subconjunctival space and forming a bleb. Reportedly, the most common cause of unsuccessful trabeculectomy surgery is subconjunctival scarring of the filtration bleb, which leads to subconjunctival fibrosis [1,2]. The findings of a large prospective randomized trial showed that a single application of mitomycin C (MMC) or 5-flurouracil (5-FU) during trabeculectomy surgery greatly improves the surgical results; i.e., the prolonged bleb persistence and IOP reduction via strong suppression of fibroblast proliferation [3]. However, their application also increases the risk of complications such as a thin bleb, bleb infection, and infectious endophthalmitis at the late phase [4,5,6].

In this present study, we investigated transforming growth factor β (TGF-β), which is known to have three isoform types in humans; i.e., β1, β2, and β3. Isoforms β1 and β2 are known to greatly stimulate the dermal scarring response [7,8]. β2 is the mainly expressed ocular isoform, and is identified in both normal and diseased eyes [9,10]. The conjunctival scarring response in trabeculectomy surgery is thought to be affected by the passage of the aqueous humor including growth factors such as TGF-β, and subconjunctival scarring post glaucoma surgery is strongly affected by cytokines (especially TGF-β in the aqueous humor) [11,12]. Compared with other growth factors, TGF-β2 is reportedly dominant in the aqueous humor of glaucoma patients [13,14]. The TGF-β family is the main stimulator leading to conjunctival scarring post trabeculectomy, and various cells, such as fibroblasts and macrophages, can secrete them [15]. It was previously reported that TGF-β2 could increase α-smooth muscle actin (α-SMA) expression and the transdifferentiation of fibroblasts in conjunctiva to myofibroblasts [16]. In another previous study, the authors’ findings revealed that bleb failure post trabeculectomy primarily occurred due to the excessive accumulation of collagen in the subconjunctival space, and that high activity of TGF-β was associated with scarring [17]. Numerous studies have reported that the expression of TGF-β activates the proliferation by human Tenon’s fibrosis, and excessive production of granulation tissue constituents leading to scar formation [18,19,20]. In addition, several studies have reported that TGF-β inhibitors may effectively reduce scarring by reducing TGF-β activity via neutralization with antibodies [21,22]. Subconjunctival injections of anti-TGF-β antibody, as a drug substituting for MMC, were performed in a clinical trial for the suppression of fibroblast proliferation post trabeculectomy, however, the outcome was reportedly unsuccessful [23].

Various drug delivery systems (DDSs) have been tested for sustained drug release, since it is important to prevent scarring over an extended period following glaucoma surgery. Several previous studies have focused on subconjunctivally implanting DDSs to provide a sustained release of antiproliferative drugs over an extended time period post glaucoma surgery [24,25,26]. Most of those studies reported that these DDSs maintained IOP reduction and prolonged bleb persistence to the same degree as the conventional application of MMC and 5-FU, while significantly reducing their toxicity. However, most of those DDSs have yet to obtain successive results in the treatment of glaucoma patients [25].

Gelatin hydrogel (GH), a biodegradable material developed in Japan, has reportedly been used as a DDS for bioactive proteins in other medical fields [27]. Various growth factors gradually released from GH have been effective for therapy of various tissues [28,29]. In addition, GH has been applied to clinical therapies, such as for severe skin lesions complicating autoimmune vasculitis syndromes, peripheral arterial disease, and severe ischemic limb pain, and was found to be both safe and effective [30,31]. In the field of ophthalmology, GH impregnated with basic fibroblast growth factor has reportedly been used to induce experimental models of subretinal or corneal neovascularization [32,33].

We previously reported the possibility of using GH containing chymase inhibitor and GH containing MMC for longer-term maintenance of filtering blebs and IOP reduction by the prolonged suppression of subconjunctival scarring [34,35]. In this present study, we investigated the effect of a sustained release of anti-TGF-β antibody from GH in a canine glaucoma surgery model for IOP reduction and the effect on tissue in comparison with the application of GH alone.

## 2. Results

### 2.1. Verification of Anti-TGF-β Antibody in GH

**Figure 1:**
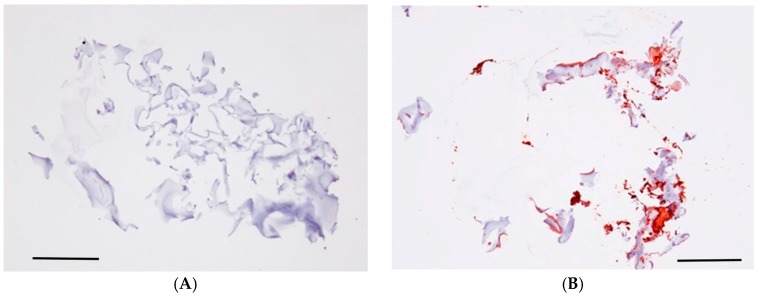
Gelatin hydrogel (GH) containing anti-transforming growth factor β (TGF-β) antibody. GH soaked overnight in phosphate-buffered saline (PBS) (**A**) did not show a positive staining image by immunostaining, however, we were able to verify a wide range of positive staining images (red) at sections of sliced GH with anti-TGF-β antibody (**B**) overnight. Scale bars: 500 μm.

Goat anti-Chicken IgY (H + L) secondary antibody was utilized to detect anti-TGF-β1-2 antibody. GH soaked overnight in phosphate-buffered saline (PBS) did not show a positive staining image by immunostaining, however, we were able to verify a wide range of positive staining images at sections of sliced GH with anti-TGF-β antibody overnight (Figure 1).

### 2.2. IOP Change

**Figure 2:**
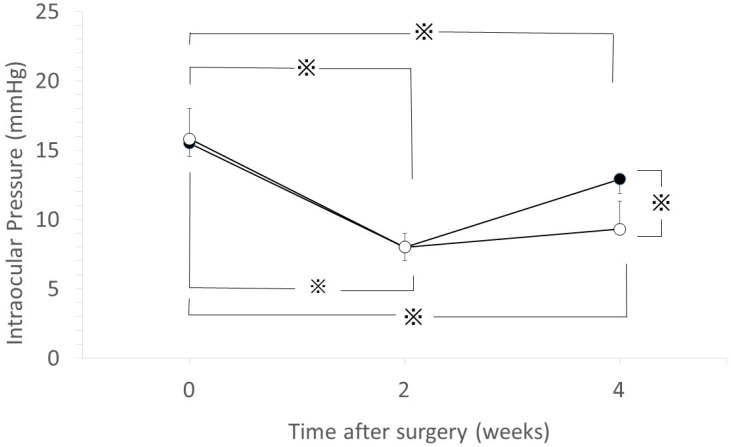
The effects to intraocular pressure (IOP) change by GH containing anti-TGF-β antibody. IOP changes in the GH-TGF-β group (○) and in the GH group (●). Data are shown as the mean ± SD of 14 beagles. (※ *p* < 0.05, unpaired *t*-test). At 4-weeks postoperative, IOP once again began to increase in the GH group, however, IOP reduction was maintained in the GH-TGF-β group (*p* < 0.05, repeated measures ANOVA).

The initial IOP values (mean ± SD) were 15.9 ± 0.7 mmHg in the GH containing anti-TGF-β antibody group (GH-TGF-β group) and 15.5 ± 0.8 mmHg in the GH alone group (GH group). The IOP values at 2-weeks postoperative were 8.1 ± 0.4 mmHg in the GH-TGF-β group and 8.0 ± 0.4 mmHg in the GH group. The IOP values at 4-weeks postoperative were 9.4 ± 0.7 mmHg in the GH-TGF-β group and 12.9 ± 0.7 mmHg in the GH group. In the eyes in both groups, IOP was found to be significantly reduced at 2- and 4-weeks postoperative (*p* < 0.05, unpaired *t*-test, Figure 2). Although there was no significant difference in IOP between the eyes in both groups at 2-weeks postoperative, IOP at 4-weeks postoperative was significantly lower in the GH-TGF-β group than in the GH group (*p* < 0.05, unpaired *t*-test). At 4-weeks postoperative, IOP once again began to increase in the GH group, however, IOP reduction was maintained in the GH-TGF-β group (*p* < 0.05, repeated measures ANOVA).

### 2.3. Bleb Score

**Figure 3:**
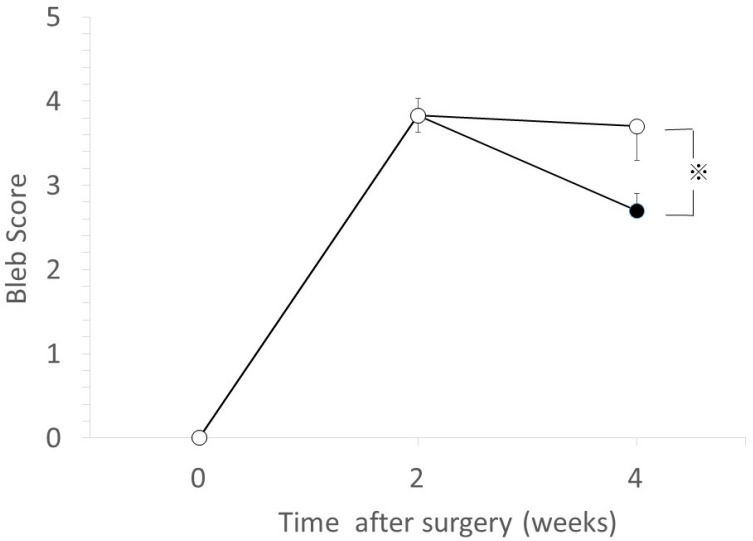
Comparison of bleb scores. Bleb score changes in the GH-TGF-β (○) group and in the GH group (●). Data are shown as the mean ± SD for 14 beagles. The bleb score at 4-weeks postoperative was significantly higher in the GH-TGF-β group than in the GH group (※ *p* < 0.05, Mann–Whitney *U*-test).

The bleb scores at 4-weeks postoperative were 3.7 ± 0.2 (mean ± SD) in the GH-TGF-β group and 2.7 ± 0.4 in the GH group; i.e., the bleb score was significantly higher in the GH-TGF-β group than in the GH group (*p* < 0.05, Mann–Whitney *U*-test, Figure 3).

### 2.4. Subconjunctival/Scleral Area Ratio

**Figure 4:**
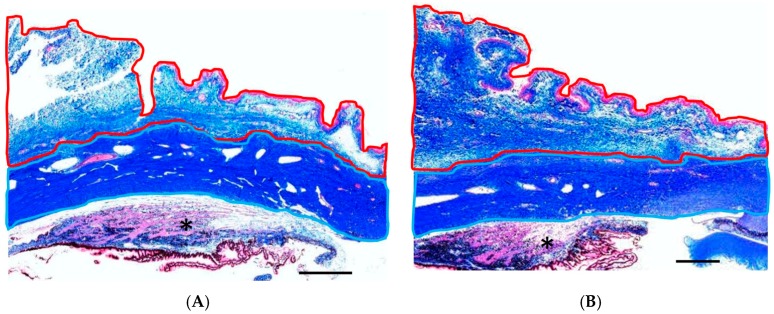
Subconjunctival/scleral area ratio. Representative photomicrographs of the sections, posterior to the sclerectomy area, obtained from the eyes treated in the GH-TGF-β group (**A**) and GH group (**B**) at 4-weeks postoperative and stained with azan stain. Collagen fiber is stained with blue. The subconjunctival area and the scleral area are surrounded by red and light-blue lines, respectively. *: ciliary body. Scale bars: 1 mm.

As shown in Figure 4, the subconjunctival area in the GH-TGF-β group was less thickened compared with that in the GH group. The ratio of subconjunctival area to scleral area was significantly lower in the GH-TGF-β-group eyes than in the GH-group eyes (*p* = 0.001, unpaired *t*-test, Table 1).

### 2.5. Vimentin-Positive Cells

**Figure 5:**
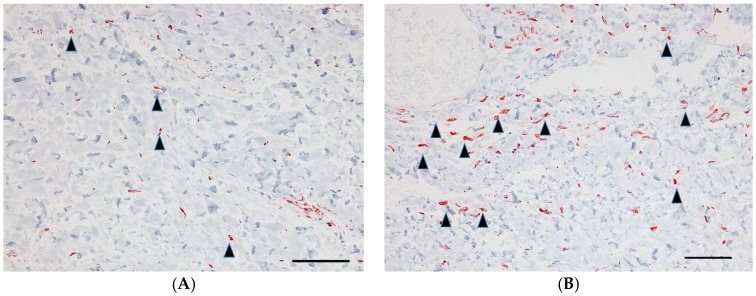
Vimentin-positive cells in the subconjunctival lesion. Representative immunohistochemical staining images of the section for vimentin in the eyes in the GH-TGF-β group (**A**) and those in the GH group (**B**). Vimentin-positive cells are indicated by black arrowheads. Scale bars: 100 μm.

A lower number of vimentin-positive cells (fibroblasts stained with anti-vimentin antibody) was found in the GH-TGF-β-group eyes than in the GH-group eyes (Figure 5). The densities of fibroblasts in the lesion were significantly higher in the GH group compared with those in the GH-TGF-β group (*p* = 0.01, Student’s *t*-test, Table 1).

### 2.6. TGF-β Antibody-Positive Cells, Proliferative Cell Nuclear Antigen (PCNA)-Positive Cells, and Mast Cells

**Figure 6:**
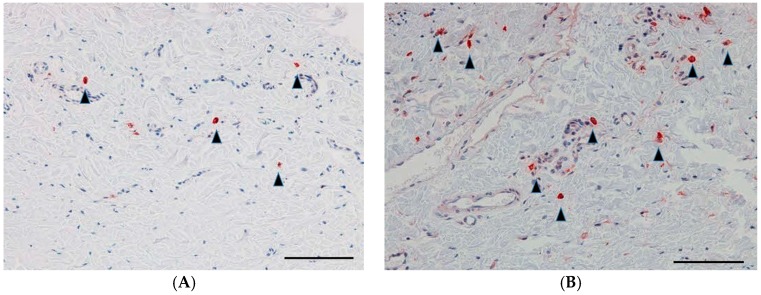
TGF-β-positive cells in the subconjunctival lesion. Representative immunohistochemical staining images of the section for TGF-β antibody-positive cells in the GH-TGF-β-group eyes (**A**) and in the GH-group eyes (**B**). TGF-β-positive cells are indicated by black arrowheads. Scale bars: 100 μm.

**Figure 7:**
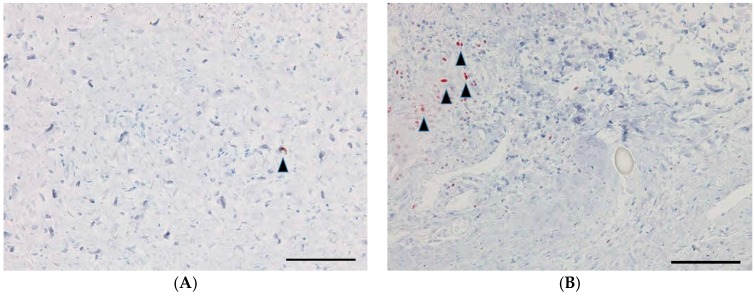
PCNA-positive cells in the subconjunctival lesion. Representative immunohistochemical staining images of the section for PCNA-positive cells in the GH-TGF-β-group eyes (**A**) and in the GH-group eyes (**B**). PCNA-positive cells are indicated by black arrowheads. Scale bars: 100 μm.

**Figure 8:**
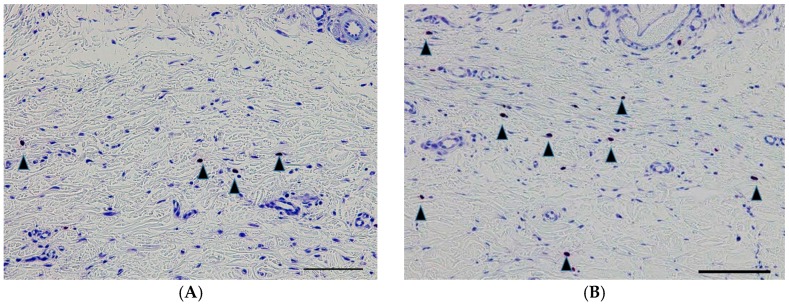
Mast cells in the subconjunctival lesion. Representative photomicrographs of the sections obtained from eyes in the GH-TGF-β group (**A**) and the GH group (**B**) and stained with Toluidine blue stain. Mast cells are indicated by black arrowheads. Scale bars: 100 μm.

The numbers of TGF-β-positive cells, PCNA-positive cells, and mast cells were also significantly lower in the GH-TGF-β group compared to the GH group (*p* = 0.04, *p* = 0.03, and *p* = 0.01, respectively, Student’s *t*-test; Figure 6, Figure 7 and Figure 8, Table 1).

## 3. Discussion

To the best of our knowledge, there have been no previous studies regarding the use of GH or other DDSs to release anti-TGF-β antibody in glaucoma surgery. Thus, this is the first experimental study regarding the effects of extended released anti-TGF-β antibody on wound healing in an experimental glaucoma filtration surgery model.

As mentioned in the Materials and Methods section, we prepared GH containing anti-TGF-β antibody. To confirm that the GH contains anti-TGF-β antibody, immunostaining was performed. We verified the existence of anti-TGF-β antibody activity in GH, indicating that GH embedded underneath the conjunctiva can release the anti-TGF-β antibody.

In this present study, we used our previously described [34,35] simple sclerotomy as a filtration surgery model to evaluate the effects of drugs or DDSs on IOP, bleb formation, and histological changes. In this model, the scleral flap and suturing flap associated with conventional trabeculectomy were not made. In order to precisely evaluate the effects of drugs in the surgery model, the same outward aqueous flow is required in all experimental eyes. However, controlling suture tightness to obtain the same outward aqueous flow is very difficult. Therefore, in this present study, we used the simple sclerotomy, as we deemed it to be the most appropriate filtration surgery model.

Our findings showed that IOP was significantly reduced at 2- and 4-weeks postoperative in both the GH group and the GH-TGF-β group. However, IOP once again began to increase at 4-weeks postoperative in the GH group, while IOP reduction was maintained in the GH-TGF-β group. This IOP change in the GH group is similar to the findings in our previously reported experiment [34,35] using the same glaucoma filtration surgery model and DDS, whereas IOP in the GH-TGF-β group was lower at 4-weeks postoperative, thus suggesting that anti-TGF-β antibody released from GH maintains IOP reduction. The bleb score at 4-weeks postoperative was significantly higher in the GH-TGF-β group than in the GH group. Together with abovementioned results, anti-TGF-β antibody from GH maintained the filtration bleb, and resulted in prolonged IOP reduction. To investigate the mechanism of the maintained bleb formation, we performed histological experiments. The ratio of subconjunctival area to scleral area was significantly lower in the GH-TGF-β-group eyes than in the GH-group eyes, thus suggesting that the above-described bleb formation in the GH-TGF-β group resulted from the inhibition of cell proliferation post glaucoma surgery in the bleb. To confirm this hypothesis, immunohistological analysis was performed. The densities of fibroblasts in the lesion were significantly higher in the GH group than in the GH-TGF-β group. The numbers of TGF-β-positive cells, PCNA-positive cells, and mast cells were also significantly lower in the GH-TGF-β group than in the GH group. Those results thus verified our hypothesis.

The findings of a previous clinical study showed no significant IOP reduction via the injection of anti-TGF-β antibody post filtration surgery [23]. The difference between the results in that study and those in this present study is whether or not a DDS was used. The injected antibody alone was washed out in the early stage due to the effect of filtration in trabeculectomy in these injection methods, whereas the sustained release of the antibody from GH was more effective to maintain bleb formation and IOP reduction. In a previous study [29], it was reported that the sustained release of TGF-β from GH enhanced the activity of bone regeneration. In that study, the authors employed a rabbit model with a calvarial defect and applied the GH containing TGF-β to the rabbit skull defect. As a control, PBS with TGF-β was employed. The authors then compared the bone mineral density (BMD) at the skull defect of the rabbit after treatment with GH containing TGF-β and PBS with TGF-β. In that study, it was described that GH containing TGF-β enhanced the BMD of the skull defect to a significantly higher extent than PBS with TGF-β. The authors also compared the release of TGF-β via subcutaneous implantation of GH containing TGF-β, and injection with TGF-β into the back of a mouse. The findings illustrated that TGF-β was retained by the implantation of GH containing TGF-β for longer time periods than TGF-β injection, and that free TGF-β disappeared from the injected site within one day. These findings indicated that the sustained release of TGF-β from GH was necessary to effectively enhance its osteoinductive function.

In the present study, we utilized GH as a DDS to spontaneously release anti-TGF-β antibody to obtain an extensive effect of anti-fibrosis in the subconjunctival area. Previous studies have shown that the controlled release of drugs over a time range of five days to three months was possible via the use of GH [36], and that the controlled release was effective for regenerative therapy of various tissues [29].

We previously reported the possibility of GH application as a new DDS to obtain a longer-term maintenance of filtering blebs [34,35]. In addition, the findings in those two reports demonstrated that the implantation of MMC-loaded GH had almost the same effects on IOP and bleb formation as the application of MMC alone in a canine filtration surgery model, and that GH containing chymase inhibitor made the period of filtration bleb formation and IOP reduction longer by decreasing cell proliferation. Taken together, this DDS is worthy of further investigation to improve the postoperative success rate of glaucoma surgery.

It should be noted that there were several limitations in the present study. First, the effect of the anti-TGF-β antibody might be different in the human eye. Thus, the toxicity of the antibody and GH DDS should be verified in primates in the future. Second, longer-term observation of the effects and toxicities of anti-TGF-β antibody might be needed. Although a 4-weeks observation period is effective for examining strong and dynamic scarring reaction in the early stage, a 3-month or greater observation period is much more informative to predict the long-term outcome and is necessary to determine whether this method can be clinically utilized. Third, conventional MMC application was not employed as a negative control, so that data is required in a future study. Fourth, since the simple sclerectomy used in the study might have a different effect on TGF-β expression around the flap site, careful interpretation from this experiment is necessary. In addition, the most effective dosage of anti-TGF-β antibody for use in GH for glaucoma surgery should be investigated.

## 4. Materials and Methods

### 4.1. Verification of Anti-TGF-β Antibody in GH

Anti-TGF-β1-2 antibody (Polyclonal Chicken IgY) was purchased from R&D Systems, Inc. (Minneapolis, MN, USA). Preparation of the GH containing the anti-TGF-β antibody was as follows: anti-TGF-β neutralizing antibody solution was produced by diluting with physiological saline, and a 5 × 5 × 1.5 mm block of freeze-dried GH (MedGel PI5^®^; Wako Chemical, Tokyo, Japan) was then soaked in the 0.1% anti-TGF-β antibody solution overnight at 4 °C. The GH preparation method used in this present study was the same as that previously described [34,35].

In order to verify that the anti-TGF-β antibody was trapped and existent in the GH, we also fixed the GH with Carnoy’s Solution (Muto Pure Chemicals Co., Ltd., Tokyo, Japan) to prepare a paraffin block. Furthermore, the GH was soaked in physiological saline overnight and compared as a negative control. All of the GH sections obtained from the above-described paraffin blocks were incubated with goat anti-Chicken IgY (H + L) secondary antibody, biotin conjugate (Thermo Fisher Scientific, Inc., Waltham, MA, USA) for 30 min at room temperature, followed by incubation with avidin-biotin-peroxidase complex (LSAB 2 Kit/HRP; Dako Japan, Kyoto, Japan) for 30 min to identify TGF-β-positive cells.

### 4.2. Animals and IOP Measurement

This experimental protocol was approved by the Committee of Animal Use and Care of Osaka Medical College (No. 28008). This study involved 14 eyes of 14 beagles purchased from Japan SLC, Inc. (Hamamatsu, Japan). The beagles were fed regular canine food, had constant free access to tap water, and were housed in an air-conditioned room at approximately 23 °C and 60% humidity with a 12-h light–dark cycle. All of the animal experiments were conducted in accordance with the ARVO Statement for Use of Animals in Ophthalmic and Vision Research. The IOP measurements were gauged via the use of a calibrated pneumatonometer (Model 30 Classic; Medtronic Solan, Jacksonville, FL, USA) under general anesthesia with intravenous injection of pentobarbital sodium (35 mg per kg body weight) in a front-facing position.

### 4.3. Glaucoma Filtration Surgery Model

For the glaucoma filtration surgery model, the beagles were anesthetized with pentobarbital sodium as described above. Briefly, a control suture was first fixed to the cornea using 8-0 Vicryl^®^ (Ethicon US, LLC., Dallas, TX, USA) suture. Next, a 10-mm fornix-based flap of conjunctiva and the Tenon’s capsule (5 mm in length) was made as previously described [34,35], and hemostasis was then performed. After a 3 × 1 mm scleral portion was removed at the limbus, peripheral iridectomy was performed. The conjunctiva was closed using a 10-0 nylon suture. After surgery, the appropriate amount of 3 mg/g ofloxacin was applied to the eye.

### 4.4. Experiment Protocol

At the end of the glaucoma filtration surgery described above, a 5 × 5 mm block of GH-TGF-β group (*n* = 7) or GH group (*n* = 7) was surgically implanted under the conjunctiva before closing the conjunctiva. In each dog, IOP and bleb score was assessed every 2 weeks for 1-month postoperative. After the final measurement, a dog was killed with a lethal dose of KCl intracardially injected and ophthalmectomy was performed, followed by soaking in 4 °C saline solution. Then, we identified the bleb area by a marked 10-0 nylon suture and excised the area by 10 × 5 mm including conjunctiva, subconjunctival tissue, and sclera and performed the following histological examination.

### 4.5. Bleb Scores

Blebs were examined via slit-lamp microscopy and graded according to the definition previously reported by Perkins et al. [37], reflecting increasing bleb height and size as follows: Score 1: minimally high conjunctiva thickening without swelling; Score 2: mild swelling present; Score 3: elevated bleb covering an area equivalent to 2–3 clock hours of the eye; and Score 4: greatly elevated bleb covering an area equivalent to more than 4 clock hours of the eye. A score of 0 indicated no observed bleb.

### 4.6. Histological Examination

Conjunctival and scleral tissue specimens were prepared for histologic analysis after fixation for 24 h with Carnoy Solution (Muto Pure Chemicals Co., Ltd., Tokyo, Japan) and embedded in paraffin. Next, 5-μm-thick sections were cut and mounted on silanized slides (Dako Japan), and then deparaffinized with xylene and a series of graded ethanol. The change in thickness of the conjunctiva and subconjunctival tissue was investigated via the ratio of subconjunctival area to scleral area stained with Hematoxylin-Eosin and Azan-Mallory. Mast cells were stained with Toluidine Blue for identification. The ratio of subconjunctival area to scleral area was then calculated (MacSCOPE Ver 2.2; Mitani Corporation, Fukui, Japan). To retrieve the antigen, sections were pretreated with 10 mM citrate buffer (pH 6.0) and autoclaved for 5 min at 120 °C before immunohistochemical staining. The sections were then soaked in absolute methanol containing 3% hydrogen peroxide for 5 min at room temperature to remove endogenous peroxidase activity. To suppress nonspecific binding, the sections were incubated with Serum-Free Protein Block (X0909; Dako Japan) for 5 min. To identify the PCNA-positive cells, the sections were incubated with mouse monoclonal antibody against PCNA (PC10, M0879; Dako Japan) for 15 h at 4 °C. Then, the slides were incubated with biotin-conjugated secondary antibody (LSAB 2 Kit/HRP; Dako Japan) for 30 min after being washed in PBS. Thereafter, those sections were incubated with avidin-biotin-peroxidase complex (LSAB 2 Kit/HRP; Dako Japan) for 30 min, washed with PBS, and then incubated with 0.05% 3,3-diaminobenzidine. The slides were then washed in running water, counterstained with hematoxylin, and mounted with cover glasses. To identify the TGF-β-positive cells, the sections were incubated with Polyclonal Chicken IgY anti-TGF-β1-2 antibody (R&D Systems, Inc.) for 15 h at 4 °C. To identify the fibroblasts, monoclonal mouse anti-vimentin antibody (M0725; Dako Japan) was also used in this present study.

We counted the PCNA-positive cells, fibroblasts, mast cells, and TGF-β-positive cells at the sites where they accumulated in the subconjunctival lesions, posterior to the sclerectomy area, by use of a light microscope (number per ×100 fields). The average number of each type of cell in five randomly selected fields of implanted GH was then calculated.

### 4.7. Masked Manner

All measurements were performed by investigators (MM and SK) who were masked from identifying which eye or tissue was in the GH-TGF-β or GH group.

### 4.8. Statistical Analysis

Each measurement was expressed as the mean ± SD. Statistical comparisons for repeated measurements used repeated-measures ANOVA, followed by other tests. Bleb scores were statistically analyzed via the Mann–Whitney *U*-test. IOP change and subconjunctival/scleral area ratio were evaluated via the unpaired *t*-test. Other parameters were evaluated via the Student’s *t*-test. Differences were considered statistically significant at a *p*-value of <0.05.

## 5. Conclusions

In conclusion, the findings of this present study demonstrated that implantation of an anti-TGF-β antibody-loaded GH was more effective for the maintenance of IOP reduction and bleb formation by the sustained release of anti-TGF-β antibody. Our findings also demonstrated that it is possible to suppress the scarring effects and maintain IOP reduction and filtration bleb formation longer than is possible with only a subconjunctival injection of anti-TGF-β antibody.

## Figures and Tables

**Table 1:** Compressions of the ratio of the conjunctival area to the scleral area, and densities of fibroblasts, TGF-β-positive cells, proliferative-cell nuclear antigen (PCNA)-positive cells, and mast cells between eyes in the GH-TGF-β group and the GH group. Data are shown as the mean ± SD for 14 beagles.

Indexes	GH-TGF-β Group	GH Group	*p*-Value (Student’s *t*-Test)
Ratio of the conjunctival area to the scleral area	1.0 ± 0.1	2.4 ± 0.1	0.001
Density of fibroblasts, per mm^2^	27.8 ± 8.6	67.6 ± 18.7	0.01
Density of TGF-β-positive cells, per mm^2^	9.8 ± 1.5	18.2 ± 3.3	0.04
Density of PCNA-positive cells, per mm^2^	4.2 ± 3.2	14.4 ± 6.0	0.03
Density of mast cells, per mm^2^	7.2 ± 1.6	13.8 ± 2.0	0.01
